# Pseudorabies virus as vaccine vectors for delivering foreign antigens: methodology and application

**DOI:** 10.3389/fimmu.2026.1997723

**Published:** 2026-10-29

**Authors:** Xiao-Xiao Tian, Yong-Bo Yang, Tong-Qing An

**Affiliations:** 1 State Key Laboratory of Animal Disease Control and Prevention, Harbin Veterinary Research Institute, Chinese Academy of Agricultural Sciences, Harbin, China; 2 China Animal Health and Epidemiology Center, Qingdao, Shandong, China; 3 Heilongjiang Provincial Key Laboratory of Veterinary Immunology, Harbin Veterinary Research Institute, Chinese Academy of Agricultural Sciences, Harbin, China; 4 Heilongjiang Veterinary Biopharmaceutical Engineering Technology Research Center, Harbin Veterinary Research Institute, Chinese Academy of Agricultural Sciences, Harbin, China

**Keywords:** CRISPR/Cas9, foreign gene expression, immune response, pseudorabies virus, virus-vectored vaccine

## Abstract

Pseudorabies virus (PRV) is the etiological agent of pseudorabies (PR). It belongs to the *Herpesviridae* family and has double-stranded linear DNA encoding more than 70 proteins. Many of these proteins are non-essential for viral replication and provide insertion sites for foreign genes to enable heterologous protein expression. PRV is a promising viral-vector platform for expressing foreign proteins and developing vaccines against multiple pathogens. However, no recombinant PRV-vectored vaccines are commercially available to date. Here, we summarize the biological characteristics of PRV, recombinant PRV construction methodology, foreign gene insertion sites and optimization, immune responses, and applications of recombinant PRV-vectored vaccines. This study provides insights into the development of PRV recombinant vaccines and the latest research progress, offering novel perspectives for advancing preventive medicine through vaccinology.

## 1 Introduction

Pseudorabies (PR), also known as Aujeszky’s disease, is a notifiable disease that causes significant economic losses to the swine industry in many countries. Pseudorabies virus (PRV), also known as *Suid alphaherpesvirus* 1, belongs to *Varicellovirus* genus, *Alphaherpesvirinae* subfamily, within the *Herpesviridae* family (1). PRV, which lacks specific host tropism, can infect a wide variety of mammals, including pigs, sheep, dogs, foxes, tigers, bears, while pigs (including wild boars) are the only susceptible species that can survive infection and are therefore thought to be the natural host (2, 3). Infected pigs exhibit symptoms such as fever, sweating, dysphagia, weakness, and neurological disorders, with high morbidity and mortality observed, especially in piglets (4). In addition to animals, over 20 cases of human pseudorabies encephalitis have been reported, primarily in individuals engaged in pig-related activities (e.g., veterinarians, swine herders, and slaughterers). Among these cases, all patients exhibited high fever and neurological symptoms, with some developing acute retinal necrosis and blindness, and a small number of patients died (5–8). The PRV genome is approximately 143 kb in size, encoding more than 70 proteins, and is characterized by two unique regions, the unique long (UL) and the unique short (US). The US region is flanked by internal repeat sequences (IRS) and terminal repeat sequences (TRS) (5, 9). PRV can be classified into two genotypes: strains isolated in Eastern and Southern Europe, Asia, Latin America, and Africa, all belong to genotype 1, whereas genotype 2 strains are found almost exclusively in China (5, 10). PRV was first identified in Hungary in 1902 and has been globally distributed. Due to increased control efforts and the strict implementation of national eradication programs, including large-scale vaccination with gE-deleted vaccines and the so-called DIVA (“differentiating infected from vaccinated animals”) strategy, countries such as the United States, Canada, and New Zealand have successfully achieved excellent control of PRV (11).

PRV infection in China was first reported in cats in 1947 and was subsequently documented in cattle, swine, and other animals (12). To control PRV, the Bartha-K61 strain, the first PRV vaccine, was imported from Hungary to China in 1979. The Bartha-K61 has played a key role in the prevention of PRV in China. However, a PRV variant emerged in the swine herds vaccinated with the Bartha-K61 strain in 2011 (13–15). The protective efficacy of the Bartha-K61 vaccine against PRV variants has attracted considerable attention. Pigs and sheep immunized with Bartha-K61 were challenged with the PRV variant HeN1 strain. The results showed that HeN1-challenged pigs exhibited symptoms such as decreased appetite and fever, without any fatalities. However, among the four HeN1-challenged sheep, two displayed clinical symptoms and died (16). Moreover, another study found that Bartha-K61 immunization did not confer complete protection in sheep against the PRV variant TJ strain (15). These studies indicate that the Bartha-K61 vaccine cannot provide complete protection against PRV variants. Recently, with a deeper understanding of the biological functions of PRV-encoding genes, commercially engineered vaccines based on PRV variant strains have been developed. For instance, the gE gene-deleted inactivated vaccines HN1201 and HNX-12 were licensed in 2019 and 2021, respectively (17, 18). However, it has been noted that PRV infection can cause immunosuppression in pigs and increase susceptibility to other pathogens (19, 20). Therefore, recombinant vaccines are important for producing a specific immune response against a target virus.

Among the various types of recombinant vaccines, PRV vector-based vaccines have the following advantages: (i) Safety and effectiveness: Existing live vaccines such as Bartha-K61, which have been used worldwide for decades, are both safe and effective. The United States, New Zealand, and several European countries have eradicated PR using the Bartha-K61 vaccine (11, 21). (ii) Large genome size for insertion: The large size of the PRV genome allows the insertion of several kilobases of foreign DNA sequences without affecting viral replication or production (22). Therefore, PRV has often been used as a vector for the development of multivalent vaccines expressing major antigens against different swine pathogens (23). (iii) Immune response: recombinant PRV (rPRV) is believed to induce both humoral and cell-mediated immunity, which are critical attributes of a vaccine. (iv) Rapid production: PRV vector-based vaccines can be rapidly produced for emerging pathogens or mutant strains by introducing the pathogen protein-encoding DNA, and a high viral titer can reduce the cost of vaccine production. Although PRV vector-based vaccines have many advantages, they also have certain disadvantages. For example, some rPRV vaccines have issues such as low expression levels of foreign proteins and instability. In addition, prior immunity against the vector may interfere with vaccine replication when the same viral vector is used repeatedly, potentially reducing the efficacy of booster vaccinations. In this review, we summarize the construction strategies, problems, and solutions for optimizing and improving rPRV vaccines.

## 2 Literature search strategy

A comprehensive literature search was performed in PubMed, supplemented by the China National Knowledge Infrastructure (CNKI) for Chinese-language publications. Search terms combined ‘pseudorabies virus’ OR ‘Suid alphaherpesvirus 1’ with ‘vector vaccine’, ‘recombinant’, ‘foreign gene expression’, ‘gene-deleted vaccine’, ‘immune response’, or specific pathogen names. Registration information for licensed vaccines was verified against announcements of the Ministry of Agriculture and Rural Affairs of China and the China Institute of Veterinary Drug Control (IVDC). Studies were included if they reported the construction, immunogenicity, or protective efficacy of recombinant or gene-deleted PRV vaccines. Reference lists of retrieved articles and relevant reviews were screened manually to identify additional eligible studies.

## 3 Construction of rPRV vaccines

### 3.1 Construction of rPRV

The use of PRV as a vector for expressing foreign proteins was first reported in 1986 (24). Subsequently, Whealy et al. constructed an rPRV expressing the PRV gIII (now known as gC) - human immunodeficiency virus type 1 envelope fusion protein, pioneering the development of PRV-based live vector vaccines (25). To develop a safe PRV vector vaccine for pigs, virulence genes of PRV, such as *TK* (26), *gE/gI* (27), were identified, and the functions of other proteins have also been summarized (9). In early studies, the genome of herpesviruses was manipulated by exposing infected cells to various chemical, physical, or biological conditions (28, 29), which is not site-specific. In recent decades, scientists have used several methods for manipulating the PRV genome, such as homologous recombination, Cre-loxP, and CRISPR/Cas9 (clustered regularly interspaced short palindromic repeats-associated protein 9), which have contributed to our understanding of PRV pathogenesis and the development of novel vaccines and antiviral drugs.

#### 3.1.1 Homologous recombination

Homologous recombination is a classical method for PRV modification. This involves cloning the gene into a plasmid transfer vector and flanking it with PRV sequences that directly recombine at the desired sites. Currently, two predominant transfection methods are used for homologous recombination in mammalian cells: transfecting the transfer vector and parent virus genome simultaneously or after infection with the virus. The transfer vector and replicating PRV genome then undergo homologous recombination, allowing the insertion of the foreign gene into the PRV genome, resulting in the generation of recombinant viruses (**Figure 1a**). Using this method, an rPRV expressing the E2 protein of classical swine fever virus (CSFV) was successfully constructed (30). Although effective for deletion, substitution, or alteration of viral genome sequences, this method has certain limitations, such as the extremely low efficiency of viral recombination (ranging from 1 in 10^6^ to 1 in 10^7^) (31). The bacterial artificial chromosome (BAC) system is another strategy used to facilitate the insertion of foreign genes into the PRV genome. The BAC system consists of three steps: (i) Construction of a transfer vector by cloning the foreign gene, flanked by homologous PRV sequences, into the pBAC-loxP plasmid; (ii) Co-transfection of the PRV genomic DNA together with the transfer vector into permissive cells to generate recombinant BAC clones; and (iii) Screening and isolation of the recombinant BAC clones by using selectable markers encoded on the vector. Red/ET recombination technology is frequently used for BAC modification in *Escherichia coli* (32). A *gD*-and *gC*-substituted PRV mutant based on Bartha-K61 (as the backbone) and the AH02LA strain (as template for *gD* and *gC* genes) was constructed using BAC technology, demonstrating growth properties equivalent to those of the parental strain in ST cells (33). In fact, both strategies, spontaneous homologous recombination within host mammalian cells and Red/ET recombination at the BAC level in *Escherichia coli* (34), have been widely applied.

**Figure 1:**
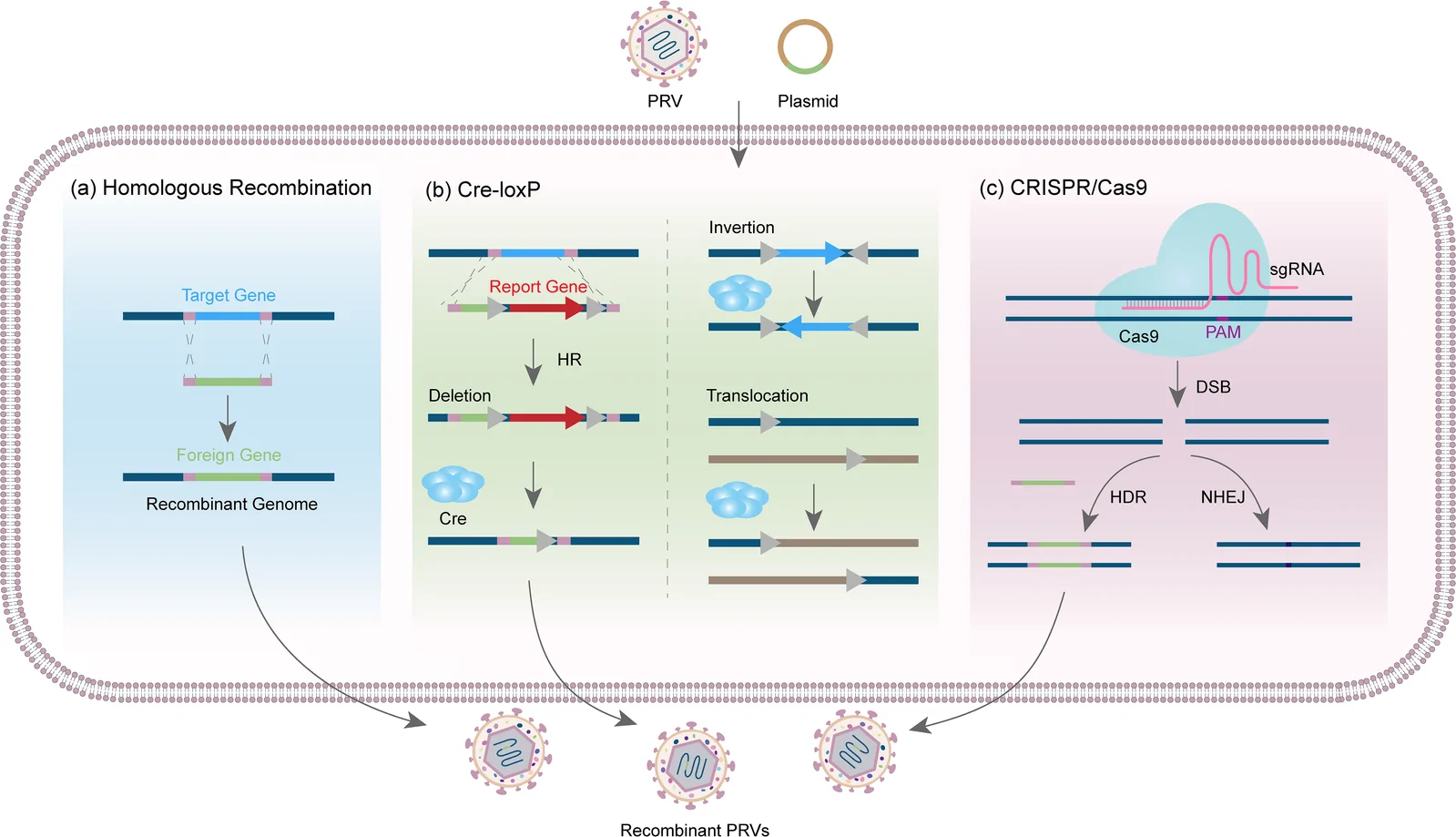
Schematic diagram illustrating the construction of PRV-vectored vaccines. **(a)** Homologous recombination. Recombination sequences (pink boxes) are included in a plasmid transfer vector to direct integration of foreign DNA (green box) into the viral genome. Homologous recombination takes place between transfer vector and PRV genome. **(b)** Cre-loxP system. Cre recombinase can recognize the loxP site and promote gene recombination. LoxP is a unique gene sequence from the P1 phage, which guides the Cre to target sites of the genome. The Cre-loxP system can produce four types of gene modification, including deletion, inversion, translocation, and cassette exchange. **(c)** CRISPR-Cas9 system. The Cas9 protein is directed to the target site by a gRNA (guide RNA) and cleaves the double strand next to the protospacer adjacent motif sequence to produce double-strand breaks (DSBs). In response to DSBs, cellular DNA repair processes result in random insertions or deletions at the cleavage site through homology-directed repair or non-homologous end joining.

#### 3.1.2 Cre-loxP

The Cre-loxP-mediated recombination system, a derivative of P1 phage, is another powerful gene editing tool that has become a mainstay of genetic and cell biology research (35). This system contains two integral components: the loxP site, which is composed of two 13 bp reverse repeats, an 8 bp asymmetric spacer, and the Cre recombinase, which selectively identifies and acts on the loxP site. The Cre-loxP localization recombination system acts via site-specific recombination using three principal mechanisms. When two loxP sites are oriented in the same direction, recombination results in the deletion of the DNA fragments between them. Conversely, Cre-mediated recombination inverts DNA fragments between loxP sites in opposite directions. Moreover, if there are two loxP sites on different molecules, such as a plasmid with one loxP site and a chromosome with another, the plasmid DNA can be integrated into the loxP site on the chromosome using Cre recombinase (**Figure 1b**). This targeted recombination capability renders the Cre-loxP system exceptionally useful for the precise manipulation of loxP-flanked DNA elements in the construction of rPRV. For example, using both homologous and Cre-loxP recombination, an rJS-2012-BAC recombinant virus carrying a BAC insertion downstream of the open reading frame (ORF) of *gG* was successfully constructed (36). Similarly, a PRV strain deleting the *US3* gene, denoted as PRV Δ*gE/TK/US3*, was generated from the PRV Δ*gE/TK* strain using the same recombination method (37).

#### 3.1.3 CRISPR/Cas9

The CRISPR/Cas9 system, an adaptive immune mechanism that evolved in archaea and bacteria, has been developed as a robust tool for DNA engineering across a spectrum of organisms and for manipulating numerous DNA and RNA viruses (38–40). The CRISPR/Cas9 system consists of single guide RNA (sgRNA) and DNA endonuclease Cas9, with the former directing the latter to specific DNA sequences to cut double-stranded DNA site-specifically. In response to double-stranded breaks, cellular DNA repair processes result in random insertions or deletions at the cleavage site through homology-directed repair or non-homologous end joining (**Figure 1c**). The CRISPR/Cas9 system is easy to design because it only requires designing sgRNAs that are suitable for target sequences. Moreover, the use of multiple sgRNA pairs targeting different sites within the same gene can significantly improve the efficiency of the viral genome editing, whereas the incorporation of a linearized transfer vector can improve the recombination efficiency between the foreign gene and the viral genome (40, 41). Currently, this method is extensively utilized as a powerful tool to construct PRV recombinants with targeted deletions in the *gE*, *gI* and *TK* genes (42–44). Furthermore, the CRISPR/Cas9 system synergizes with homologous recombination to improve the recombination efficiency (31, 45). For example, the rPRV-NC56 strain was developed using CRISPR/Cas9-mediated homologous recombination technology, enabling the stable expression of GP5 and M proteins (43). Similarly, another rPRV was constructed using the same approach to express GM-CSF, GP3, and GP5 proteins derived from NADC30-like porcine reproductive and respiratory syndrome virus (PRRSV), and conferred protection against PRRSV and PRV co-infection in piglets (46). Despite numerous advantages, the CRISPR/Cas9 system has several limitations. For instance, the requirement for sgRNA sequences can pose challenges in designing guides to produce single-site mutations. Additionally, the potential off-target activity of Cas9 may lead to unexpected mutagenesis (47).

### 3.2 Insertion positions for foreign genes

PRV is a promising vector for the expression of heterologous proteins due to its ability to accommodate foreign gene insertions (**Figure 2**) (48, 49). However, ensuring the efficacy of the vaccine requires careful consideration of insertion sites. Virulence genes like *gE*, *gI*, and *TK* of PRV strains (9, 26, 27) have minimal impact on viral immunogenicity, making them highly favorable targets for foreign gene insertion. Genomic loci corresponding to the deletion of PRV virulence genes are generally suitable for the insertion of a diverse array of foreign genes (**Table 1**). For example, the rPRV strain TJ-del*gE*/*gI*-E2, constructed by substituting the gE and gI genes with an expression cassette encoding the E2 protein of CSFV, and conferred complete protection against lethal challenge with both the PRV TJ and the CSFV Shimen strains (52). In a subsequent study, the capsid (Cap) gene of porcine circovirus type 2 (PCV2) and the E2 gene of CSFV were introduced into the *TK* and *gE/gI*-deleted regions of PRV, respectively, resulting in an rPRV strain that co-expressed both CSFV E2 and PCV2 Cap proteins. This recombinant virus strain was phenotypically stable for at least 20 passages in cell culture (61).

**Figure 2:**
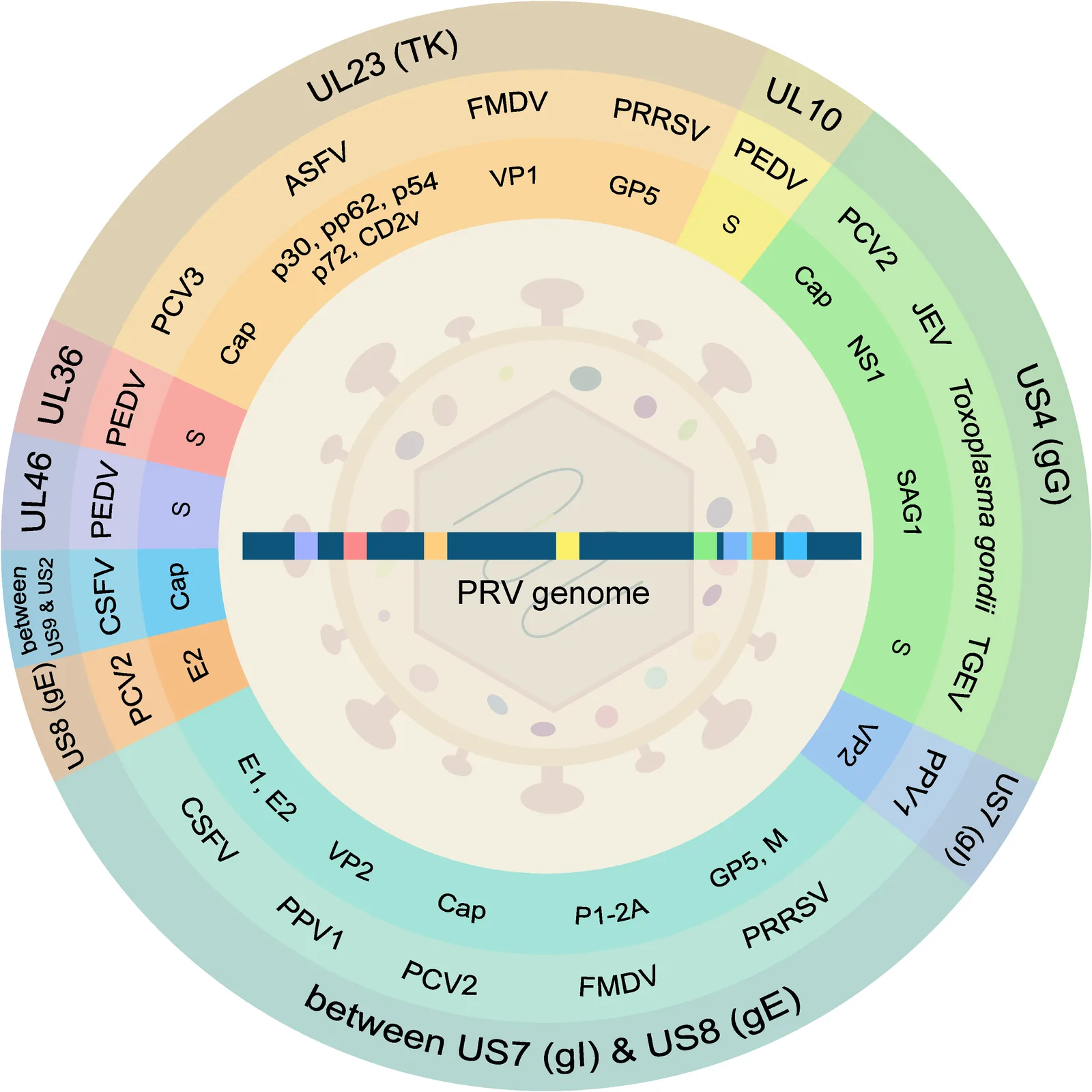
Summary of PRV vectored vaccines. Shown from the outer circle to the inside are the gene sites inserted by foreign proteins, viruses and their proteins.

**Table 1:** List of genetically modified vaccines or candidates against PRV infection.

Gene-deleted vaccines	Strain	Features	Status	Reference
Single gene deleted vaccine	HNX-12 (variant)	gE	Licensed	*
	HN1201 (variant)	gE	Licensed	(17)
Double gene deleted vaccine	HNX (variant)	TK/gE	Non-licensed	(50)
	Ea (classical)	TK/gE	Non-licensed	(51)
	TJ (variant)	gE/gI	Non-licensed	(52)
	JS-2012 (variant)	gE/gI	Non-licensed	(30)
	Ea (classical)	gG/TK	Licensed	*
	ZJ011G (variant)	gE/gI	Licensed	*
	HB98 (classical)	gG/TK	Non-licensed	(53)
Triple gene deleted vaccine	HeB12 (variant)	TK/gE/gI	Non-licensed	(54)
	TJ (variant)	TK/gE/gI	Non-licensed	(55)
	XJ (variant)	TK/gE/gI	Non-licensed	(56)
	SA125 (classical)	TK/gE/gI	Licensed	*
Quadruple-gene deletion	C (variant)	gI/gE/US9/US2	Licensed	*
	NY (variant)	TK/gE/gI/gG	Non-licensed	(57)
Quintuple-gene deletion	HN1201 (variant)	TK/gE/gI/11k/28k	Non-licensed	(44)
	XJ (variant)	TK/gE/gI/US9/US2	Non-licensed	(43)
	XJ (variant)	TK/gE/gI/US9/US2	Non-licensed	(58)
	XJ (variant)	TK/gE/gI/US9/US2	Non-licensed	(59)
	GX (variant)	TK/gI/gE/US9/US2	Non-licensed	(60)

“Licensed” refer to commercial veterinary vaccines licensed in China. “*” indicates information obtained from the China Veterinary Drug Information Network (http://www.ivdc.org.cn).

Genetic stability is crucial for live vector vaccines and may be related to the chosen insertion site. For example, one study observed that the CSFV E2 gene inserted into the *gE/gI* deletion region of PRV underwent partial deletion after 15 passages. However, when the E2 gene was introduced between *gG* and *gD*, no mutations or deletions were detected even after 20 passages (30). However, research on the impact of insertion sites on the stability of the same foreign gene is limited, and further investigation is needed to determine whether the insertion site is a key factor. Additionally, the inadequate expression of heterologous proteins can contribute to immune failure. To address this issue, foreign genes have been inserted at the gG locus, which is regulated by a strong early PRV promoter (22, 62). Moreover, the insertion of foreign genes into non-coding regions has been studied. For example, the spike (S) protein of porcine epidemic diarrhea virus (PEDV) was inserted into the *UL11-10*, *UL35–36* and *UL46/UL27* regions in the PRV AH02LA BAC strain with *TK*, *gE* and *gI* deletions and effectively expressed (48). These results provide new insights into the strategic selection of suitable foreign gene insertion sites for rPRV vaccines.

### 3.3 Promoters for foreign gene expression

An efficient expression system relies on multiple factors, notably the presence of a potent promoter within the framework of a suitable expression plasmid, which is essential for driving target gene transcription (63). Various promoters have been studied for the construction of rPRV vaccines (**Table 2**). Some, like the cytomegalovirus (CMV) IE1 and chimeric CAG promoters, are constitutively active, while others, such as the *gE*, *gG* and *gD* promoters, are regulated during viral infection. For example, multiple copies of PCV2 *ORF2* (encoding the Cap protein) were driven by the strong *gE* and *gG* promoters, ensuring robust expression of exogenous *ORF2* in the PRV vector (64). Additionally, the *gD* promoter was selected to drive the expression of the Fms-related tyrosine kinase 3 ligand (Flt3L). Consequently, a significant increase in the number of activated dendritic cells in mice immunized with HNX-*TK^−^*/*gE^−^*Flt3L was observed, as compared with those treated with HNX *TK^−^/gE^−^* (67). Additionally, stronger heterologous enhancers or promoters, such as the human CMV (HCMV) or murine CMV (MCMV) IE1 promoter/enhancer complex, have been widely used (45, 50, 68). It was found that the chimeric CAG promoter, which is composed of the HCMV enhancer and chicken beta-actin promoter, is superior to the HCMV and MCMV promoters in enhancing the expression of foreign genes in PRV (69). Furthermore, the expression efficiencies of the immediate-early cytomegalovirus (IE-CMV) and *gG*-PRV promoters were compared using PRV amplicons as vectors and enhanced EGFP as the expression marker. Both promoters were equally effective in expressing EGFP (70). However, in another study, the CSFV *E1* gene exhibited improved expression under the control of the immediate-early promoter of HCMV compared with the *gD* or *gE* promoters, leading to an enhanced immune protection effect after CSFV and PRV challenge (71).

**Table 2:** List of recombinant live attenuated vaccines against PRV infection.

Strain	Construction strategy	Target pathogens	Insert sites	Promoter	Immune model	Inoculation route	Antibody production		Efficacy	Reference
HNX-ΔTK/ΔgE-Cherry (Genotype II)	CRISPR/Cas9	PCV3/ORF2(Δ1-33aa)	TK	CMV	Mouse	IM (twice)	Induction of anti-PCV3 antibodies		Protection: ND	(50)
HB98-ΔTK/gG (Genotype II)	HR	PCV2/Cap/IL-18	gG	gG Promoter	Mouse	ID (twice)	Induction of anti-PCV2 antibodies; higher than those of the PRV-ORF2 and inactivated PCV2 vaccine groups		100% protection (targeting PCV2 HN strain)	(53)
PRV-ΔTK/gE/GFP^+^ (Genotype II)	CRISPR/Cas9	PCV2/Cap	gE/gG	gE Promoter	Mouse	IM (twice)		Induction of anti-PCV2 antibodies	Protection: ND	(64)
					Pig	IM (twice)		Induction of anti-PCV2 antibodies		
TJ (Genotype II)	Fosmid Library Platform	PCV2 Cap/CSFV E2	Between gE and gI	CMV	Rabbit	IM (twice)		No detectable antigen-specific antibodies	\	(61)
					Pig	IM (twice)		No detectable antigen-specific antibodies	\
rPRV TJ-ΔgE/gI-eGFP (Genotype II)	HR	CSFV/E2	Between gE and gI	CMV	Pig	IM (twice)		Induction of anti-CSFV antibodies, but lower than C strain	100% protection (targeting CSFV Shimen strain); pig immunized with 10^6 or 10^5 TCID_50_ of rPRV TJ-ΔgE/gI-E2 show no lesions, but 2/5 pig immunized with 10^4 TCID_50_ showed mild lesions	(52)
JS-2012-ΔgE/gI (Genotype II)	HR	CSFV/E2	Between gG and gD	CMV	Pig	IM (once)		Piglets with negative maternal antibodies did not produce antibodies until 5 days after challenge; Piglets with positive maternal antibodies did not produce antibodies until 7 days after challenge	100% protection of pig with negative maternal antibodies; and 80% protection of pig with positive maternal antibodies	(30)
Fa (Genotype II)	CRISPR/Cas9	ASFV/CD2v	TK	CMV	Mouse	IM (twice)		Induction of anti-ASFV antibodies	Protection: ND	(65)
SA215 (Genotype II)	HR	PPV1/VP2	gI	CMV	Pig	IM (twice)		Induction of anti-PPV antibodies	Protection: ND	
					Primiparous gilts	IM (once)		Induction of anti-PPV antibodies	100% protection (targeting PPV SC1 strain), and 3.6% (1/28) mortality rates of fetuses	(41)
rPRV-VP2-EGFP (Genotype II)	HR	PPV1/VP2/IL-6	gE	CMV	Mouse	IM (twice)		Induction of anti-PPV antibodies	90% protection (targeting PPV HN strain)	(66)

Beyond the individual choice of promoter and insertion locus, the spatial relationship between them also affects foreign gene expression. A cassette inserted in the same transcriptional orientation as the flanking viral gene, or close to a strong early promoter such as that of gG, generally yields higher expression, whereas convergent or tandem arrangements of two strong transcription units may cause transcriptional interference, promoter occlusion or read-through, reducing antigen yield. These positional effects help explain why the same promoter-antigen combination performs differently at different loci, indicating that locus, promoter and their spatial arrangement should be optimized jointly. This principle extends to other viral vectors: in a rabies virus vector, the glycoprotein gene was duplicated, thereby altering both its copy number and genomic position. This modification increased G expression by 2.47-fold (mRNA) and 3.4-fold (protein), raised viral titers, and enhanced neutralizing antibody responses approximately two- to three-fold (72).

### 3.4 Foreign genes and their modifications

Various genes encoding antigens from animal pathogens have been successfully integrated into the PRV genome. For example, the gene encoding the Cap protein of the porcine parvovirus (PPV) was cloned into the PRV SA215 vector, leading to the self-assembly of virus-like particles (VLPs). The VLPs were similar in size and morphology to the original virions, exhibited high immunogenicity and provided protection (41). However, the integration of certain foreign genes into the PRV genome may lead to immune failure, characterized by a lack or reduction in antibody production (61). Researchers have undertaken additional modifications and optimizations regarding the quantity and type of exogenous gene insertion to improve antigenicity and expand the range of potential vaccine candidates. Multiple copies of the PCV2 ORF2 gene (two, four, and eight copies), connected via a protein quantification ratio (PQR) linker, were inserted into the PRV genome to enhance the expression level of the PCV2 immunogen. The PQR linker is a synthetic peptide sequence used to regulate and monitor the expression of each ORF2 copy in tandem arrangements. Notably, the insertion of two separate copies of the quadruple ORF2 gene cassette, each containing four tandem ORF2 sequences and integrated at distinct sites in the PRV genome, resulted in the highest yield of Cap protein (64). PQR plays a crucial role in ensuring equal expression levels across each copy of ORF2 (73). Furthermore, self-cleaving T2A and P2A peptides enable the production of individual proteins from a single transcript through ribosomal skipping, thereby minimizing the need for post-translational processing and reducing potential interference between adjacent protein products. With the introduction of the self-cleaving 2A peptide, GP5 and M proteins were expressed independently, forming VLPs of PRRSV in rPRV-NC56-infected BHK-21 cells, which subsequently induced neutralizing antibodies in the serum of rPRV-NC56-immunized mice (43). It is also noteworthy that, in addition to enabling the expression of full antigen genes, truncated expression of foreign genes or expression of a single epitope can also be effective. For example, the PCV3 Cap protein, with a truncated nuclear localization signal (NLS), was stably expressed after insertion into the PRV TK gene and successfully induced the production of specific antibodies against the Cap protein (50). Moreover, a recombinant virus co-expressing two distinct neutralizing epitopes derived from the spike proteins of transmissible gastroenteritis virus (TGEV) and PEDV was constructed, which induced neutralizing antibodies against both viruses. Additionally, the expression of foreign genes can be enhanced by regulatory elements such as the Kozak sequence (e.g., GCCACC), which promotes efficient translation initiation in eukaryotic cells (74). Furthermore, optimizing the codon usage of foreign genes to match the codon-usage preferences of the target host species is another crucial strategy for improving expression levels. Protection depends on antigen conformation, correct folding/VLP assembly, secretion, and the presentation of neutralizing rather than decoy epitopes (e.g., wild-type GP5 expressed by rPRV-GP5 failed to elicit neutralizing antibodies, whereas the epitope-modified GP5m did), and excessive expression may compromise genetic stability. Therefore, the expression of antigen should be optimized rather than maximized, and validated by challenge.

### 3.5 Cytokine as immune adjuvant

Enhancing the immune response in vaccine-immunized animals can be achieved through various strategies, including the use of vaccine adjuvants (such as oil emulsions, bacterial products, and liposomes) or by combining them with proteins encoded by immune molecules. Molecular adjuvants, distinct from traditional adjuvants, are immunomodulatory proteins delivered through plasmids, viral vectors, or genome integration, and act by engaging innate immune receptors or modulating signaling pathways. An increased understanding of the molecular mechanisms underlying both adaptive and innate immune responses has facilitated the development of molecular adjuvants for vaccine applications. These molecular adjuvants encompass a range of substances, including cytokines, co-stimulatory molecules, and pathogen recognition receptor agonists. Currently, cytokines are predominantly used in PRV recombinant vector vaccines that primarily target the innate immune response pathway (**Table 2**).

Several immune adjuvants have been used in combination with rPRV vaccines to enhance immunogenicity. For example, interleukin 6 (IL-6), a pleiotropic cytokine, is recognized for its role as an important immune adjuvant that not only induces activated B cells to produce immunoglobulins, but also promotes T cell differentiation (75, 76). Mice immunized with the rPRV-VP2-IL6 strain exhibited higher levels of antibodies against PPV than those immunized with rPRV-VP2-eGFP, underscoring the potential of IL-6 as an adjuvant (66). Previous research has confirmed that fusion proteins containing IL-6 and CpG sequences (another emerging adjuvant) not only retain the effects of the original individual proteins, but also exhibit novel or heightened abilities to regulate immune responses or cellular metabolism (77–79). Therefore, composite biological adjuvants may elicit more robust immunological responses. IL-18, as an immune adjuvant, enhances vaccine immune responses by activating natural killer cells and CD8+ T cells, boosting antigen presentation and cytokine secretion, and promoting Th1-type immune responses. It has been applied to rPRVs. A previous study demonstrated that mice immunized with PRV-Cap-IL18 generated significantly higher levels of PCV2-specific antibodies than those vaccinated with PRV-Cap alone or in combination with commercially available PCV2 vaccines (53). To further enhance the immune response, IL-18 has been investigated in combination with other adjuvants such as IL-12 (80) and IL-15 (81), both of which have shown favorable immunological effects. For example, in a previous study, PRV gB and gD proteins expressed via a baculovirus system were formulated with GM-CSF as an adjuvant, resulting in significantly enhanced antibody responses and survival in rabbits compared to the formulation with ISA 201 (82). Moreover, combining porcine GM-CSF and FliC as adjuvants significantly enhanced PCV vaccine-induced immune responses in mice and pigs, indicating their potential to improve vaccine efficacy (83). Currently, the use of GM-CSF as an adjuvant in rPRV vaccines remains unexplored and offers a promising avenue for future studies.

## 4 Immune response to rPRV vaccines

### 4.1 Immunization route

The immunogenicity of vaccines is influenced by the presentation of viral antigens, including inactivated viruses, attenuated viruses, replication-defective viruses, and adjuvanted subunit proteins. Additionally, the dosage and route of vaccine administration are pivotal factors. The most common routes of immunization include subcutaneous (SC), intradermal (ID), intramuscular (IM), and intranasal (IN) administration. Among PRV recombinant vaccines, the IM route was predominantly employed, followed by SC administration (**Table 2**). For example, mice immunized with PRV-*ORF2*-IL18 via SC administration exhibited high levels of PCV2-specific binding antibodies and neutralizing antibodies, as measured by enzyme-linked immunosorbent assay (ELISA) and serum neutralization assays, respectively (53). In contrast to injectable vaccines, mucosal immunization offers a promising strategy to overcome inherent limitations by providing frontline defense against pathogen invasion at mucosal surfaces. Mice immunized with rPRV-gE/TK/GP5m+/IL-18 using a combination of mucosal and SC immunization successfully produced neutralizing antibodies against PRRSV (84). However, further investigations are required to determine whether the combined immunization strategy elicits a superior immune response compared with individual approaches.

### 4.2 Immune response

#### 4.2.1 PRRSV

The PRRSV is the causative agent of PRRS, which is arguably the most economically significant disease to the pig industry worldwide. Its genome is composed of at least 11 ORFs which encode eight structural proteins and at least 14 non-structural proteins (NSP). Previous studies have identified multiple neutralization epitopes on major structural proteins (GP5 and M) and minor glycoproteins (GP2a, GP3, and GP4) (85, 86). Two decades ago, an rPRV expressing the GP5 protein of PRRSV (rPRV-GP5) was developed using the Bartha-K61 strain. However, piglets vaccinated with rPRV-GP5 failed to generate detectable levels of anti-GP5 antibodies or neutralizing antibodies (NAbs) (87). To enhance the protective efficacy of rPRV-GP5, a modified GP5 gene (GP5m) was synthesized by incorporating a Pan DR T-helper cell epitope (PADRE) between the neutralizing epitope and the decoy epitope (88). Subsequently, four distinct PRV recombinants, namely, rPRV-GP5, rPRV-GP5m, rPRV-GP5-M, and rPRV-GP5m-M, were constructed. Mice immunized with rPRV-GP5m-M exhibited the highest levels of PRRSV-specific NAbs and lymphocyte proliferation (89). This finding suggests that modification of the GP5 gene may offer a more effective strategy for vaccine development against PRRSV infection than the co-expression of GP5 and M proteins. Recently, a novel rPRV (rPRV-NC56) was engineered to co-express NADC30-like PRRSV GP5 and M proteins, which self-assemble into VLPs (43). rPRV-NC56 elicited significant levels of NAbs against both PRV and NADC30-like PRRSV, providing complete protection against PRV in mice. However, before its practical application, further studies are necessary to determine whether this vaccine can protect pregnant sows from reproductive failure.

#### 4.2.2 PPV1

PPV1 is a small, non-enveloped virus that causes reproductive problems in pigs. The PPV1 genome is a single-stranded DNA molecule of approximately 5 kb, encoding three structural proteins: VP1, VP2, and VP3 (90). The structural viral protein VP2 is crucial for inducing PPV-specific NAbs and confers protective immunity by self-assembling into empty capsids (91, 92). Using PRV as a vector, the gene encoding VP2 was integrated into the PRV genome, enabling VP2 proteins to self-assemble into VLPs. Piglets immunized with the recombinant virus elicited both PRV-specific and PPV-specific humoral immune responses, providing complete protection against lethal doses of PRV. However, the recombinant vaccine offered only partial protection against PPV1 in gilts (41). Another study constructed a multi-gene-deleted recombinant PRV (rPRV-VP2) based on the PRV variant HLJ8, expressing a consensus PPV1 VP2 protein. This rPRV-VP2 induced robust antibody responses and provided complete protection against both PRV and PPV1 challenges in both mice and piglets (93). To improve the immune response, a novel rPRV (rPRV-VP2-IL6) was constructed to co-express IL-6 and VP2 protein. Although rPRV-VP2-IL6 exhibited stronger immune protection in mice compared with rPRV-VP2-EGFP, it failed to confer complete protection against PPV1 (66). Therefore, further investigation into recombinant vaccines with enhanced immune efficacy is warranted.

#### 4.2.3 CSFV

CSFV, the etiological agent of classical swine fever (CSF), is a highly contagious pathogen (94). The genome of CSFV contains a single ORF, which encodes a large polyprotein that is cleaved to produce multiple structural and non-structural proteins. Among these, the E2 protein has strong immunogenicity and is commonly used in vaccine development (95). The glycoprotein E2 of CSFV is recognized for its ability to elicit protective immunity in pigs and is frequently incorporated into genetically engineered CSF vaccines (96, 97). For example, rPRV TJ-delgE/gI-E2 involves the insertion of an E2 expression cassette into the intergenic region between *gI* and *gE*. This recombinant virus has been demonstrated to be safe for pigs, successfully inducing detectable levels of anti-PRV and anti-CSFV NAbs and providing complete protection against both the PRV TJ and CSFV Shimen strains (52). This immunoprotective effect was corroborated by another study (68). In contrast to these findings, a different recombinant virus, JS-2012-Δ*gE*/*gI*-E2, was constructed by integrating the E2 expression cassette between the *gD* and *gG* genes. Although this recombinant virus effectively protected pigs against lethal challenge with the CSFV Shimen strain, seroconversion of E2-specific antibodies was not observed prior to the challenge (30). We hypothesize that this divergence in seroconversion may be attributable to the administration of a single dose of the rPRV-vectored vaccine; however, this explanation remains to be experimentally confirmed (98). In addition, one possible explanation for the reduced antibody levels is diminished secretion of the E2 protein; this has not been directly demonstrated for this construct and therefore remains a hypothesis requiring experimental validation. It has been reported that a more stable protein structure can enhance the efficiency of protein secretion through the eukaryotic secretion pathway (99). Therefore, the Rosetta tool was used to optimize the thermal stability of E2. As a result, a recombinant virus (rPRV-muta/287aaE2) was constructed, which showed a 2.97-fold increase in secretion efficiency and a 10.5-fold improvement in thermal stability of the stabilized E2 protein. Subsequent immunization of mice doubled antibody production and achieved a balanced antibody level against both subtype 1.1 and subtype 2.1d E2 (100). These findings provide a new approach for optimizing immunogens.

#### 4.2.4 PCV

To date, four species of PCVs, namely PCV1, PCV2, PCV3, and PCV4, are ubiquitous in global pig populations (101). The PCV genome encodes two ORFs, ORF1 and ORF2, with ORF2 encoding the capsid protein. Additionally, the Cap protein, a component of the Capsid, has significant immunogenic properties and is commonly targeted in vaccine development (102). The rPRV, rPRV-Δ*gE*-Cap, was constructed by inserting the PCV2 Cap gene into the *gE* gene, leading to the production of Cap protein that self-assembled into VLPs. Mice and guinea pigs immunized with purified VLPs exhibited specific antibody responses against the PCV2 Cap protein (103). Compared with rPRV expressing the Cap protein (rPRV-*ORF2*), the rPRV-*ORF2*-IL-18, which co-expresses IL-18 and the Cap protein, elicited higher titers of serum NAbs and stronger cell-mediated immune responses in mice, suggesting the potential of porcine IL-18 to enhance humoral immune responses (53). However, further research is needed to evaluate the immunological protective effects of these vaccines in pigs prior to their practical application. In a separate study, to improve the expression of the PCV2 immunogen, the PCV2 *ORF2* was connected in tandem repeats using a PQR linker to ensure equal expression of each *ORF2* gene copy. This strategy led to the successful generation of a highly effective bivalent recombinant virus, PRV-8×*ORF2*, which induced high titers of antibodies against PRV and PCV2 in both experimental pig and mouse models (64). Additionally, PRV expressing the Cap protein of PCV3 has also been studied. The PCV3 *ORF2* gene, with a truncated N-terminal NLS, was inserted into the *TK* gene, resulting in the recombinant HNX-Δ*TK*/Δ*gE*-*ORF2*. This recombinant virus successfully produced Cap-specific antibodies and activated PRV-specific cellular immune responses (50). However, it should be noted that PCV3 was only recently isolated, necessitating further evaluation to confirm the efficacy of the vaccine against PCV3 infection.

#### 4.2.5 Multi-pathogens

During intensive pig production, it is common for swine to be simultaneously infected with multiple pathogens. Coinfections with PRV and other pathogens such as CSFV, PCV2, PPV, and Foot-and-mouth disease virus (FMDV) are frequently observed and have significant implications for disease presentation (4). Between 2013 and 2018, studies in China revealed co-infection rates of 36.0% for PRRSV, 12.9% for PCV2, and 1.8% for CSFV in pigs, underscoring the urgency for multivalent vaccines that can address these concurrent challenges (20). Therefore, the development of multivalent vaccines is a strategic solution, potentially streamlining vaccination protocols and reducing the financial burden on the industry. For example, a triple-gene (TK/gE/gG)-deleted vaccine vector was developed to express chimeric PCV2b-capsid, CSFV-E2, and chimeric E^rns^-fused with bovine GM-CSF (E^rns^-GM-CSF), designated as PRVtmv+. This vaccine successfully stimulated the production of viral NAbs against PRV, PCV2b, and CSFV in vaccinated pigs. Notably, PRVtmv+ demonstrated nearly twice the level of PCV2b-specific NAbs titer after PCV2b challenge compared with the commercial Fostera Gold PCV vaccine (104). In a separate study, a recombinant virus was constructed using the PRV *TK^-^*/*gE^-^*/*LacZ^+^* mutant as the vector, co-expressing the P1-2A capsid precursor protein (the P1 capsid precursor fused to the 2A peptide) of FMDV and the VP2 protein of PPV. However, this recombinant induced only 60% of the antibody levels achieved by inactivated vaccines against FMDV or PPV, indicating that further optimization is necessary to improve its immunogenicity (105). Collectively, these findings provide compelling evidence that rPRV may serve as a promising platform for the development of novel vaccines capable of controlling multiple diseases simultaneously.

#### 4.2.6 Others

In addition to its application in viral vaccine development, PRV has also been explored as a vector system for the expression of immunogenic proteins from various bacterial and parasitic pathogens. One notable example is *Brucella suis*, a zoonotic bacterium that causes brucellosis, leading to abortions and reproductive failure in infected animals. Despite its economic and public health significance, no safe and highly efficacious vaccine is currently available. To address this challenge, an rPRV was engineered using a live PRV vaccine strain as a vector to express the Brucella BP26 protein, a diagnostic and immunogenic antigen. This construct successfully induced both humoral and cellular immune responses (106). However, further research is needed to investigate the protective efficacy of this recombinant virus and determine the bacterial load of *Brucella* following a challenge. Additionally, *Toxoplasma gondii* (*T. gondii*), another zoonotic species, infects nearly all mammals and avian species. An rPRV expressing the immunodominant surface antigen 1 (TgSAG1) of *T. gondii* has been studied but provided only partial protection (60%) against lethal challenge with the highly virulent *T. gondii* RH strain (107). This may be attributable to the complex life cycle of *T. gondii*, in which immunity against a single antigen may be insufficient to provide complete protection (108). Although there is limited research on the expression of bacterial or parasitic antigens using PRV, existing studies highlight PRV as a promising viral vector for the expression of immunogenic proteins.

### 4.3 Comparison of humoral and cellular immunity

The preceding sections summarize immune responses according to the target pathogen; here we compare the two arms of adaptive immunity systematically (**Figure 3**). In most studies the humoral arm, assessed as PRV- and pathogen-specific neutralizing antibodies and ELISA antibodies, has been the principal endpoint. Constructs expressing VLP-forming antigens (such as PCV2 Cap, PPV1 VP2 and PRRSV GP5+M), as well as those co-expressing IL-6, tend to favor this arm, with IL-6 promoting plasma-cell differentiation and antibody production (64, 66). By contrast, the cellular arm​ is evaluated less frequently and less completely; where assessed, it is reflected in cytotoxic T lymphocyte (CTL) activity, lymphocyte proliferation, Th1/Th2 cytokine profiles and NK-cell activation, and is preferentially enhanced by Th1-polarizing adjuvants such as IL-18, IL-12 and IL-15, and by antigens that are efficiently cross-presented. Several parameters shape the balance between the two arms: the antigen form (VLP versus monomeric), the promoter and consequent expression level, the cytokine adjuvant employed, and the immunization routes, with intranasal delivery additionally engaging mucosal immunity whereas intramuscular and subcutaneous injection mainly induce systemic responses. Notably, although most rPRV candidates activate both arms to differing extents, the two are rarely quantified within the same study and their relative contributions to protection are seldom dissected. Moreover, as emphasized above, regardless of whether it is humoral or cellular, robust immunogenicity does not necessarily translate into protection, so challenge data remain indispensable. Future studies should therefore evaluate humoral and cellular responses in parallel and correlate each with challenge outcomes, enabling rPRV constructs to be rationally optimized towards the arm of immunity required for a given pathogen.

**Figure 3:**
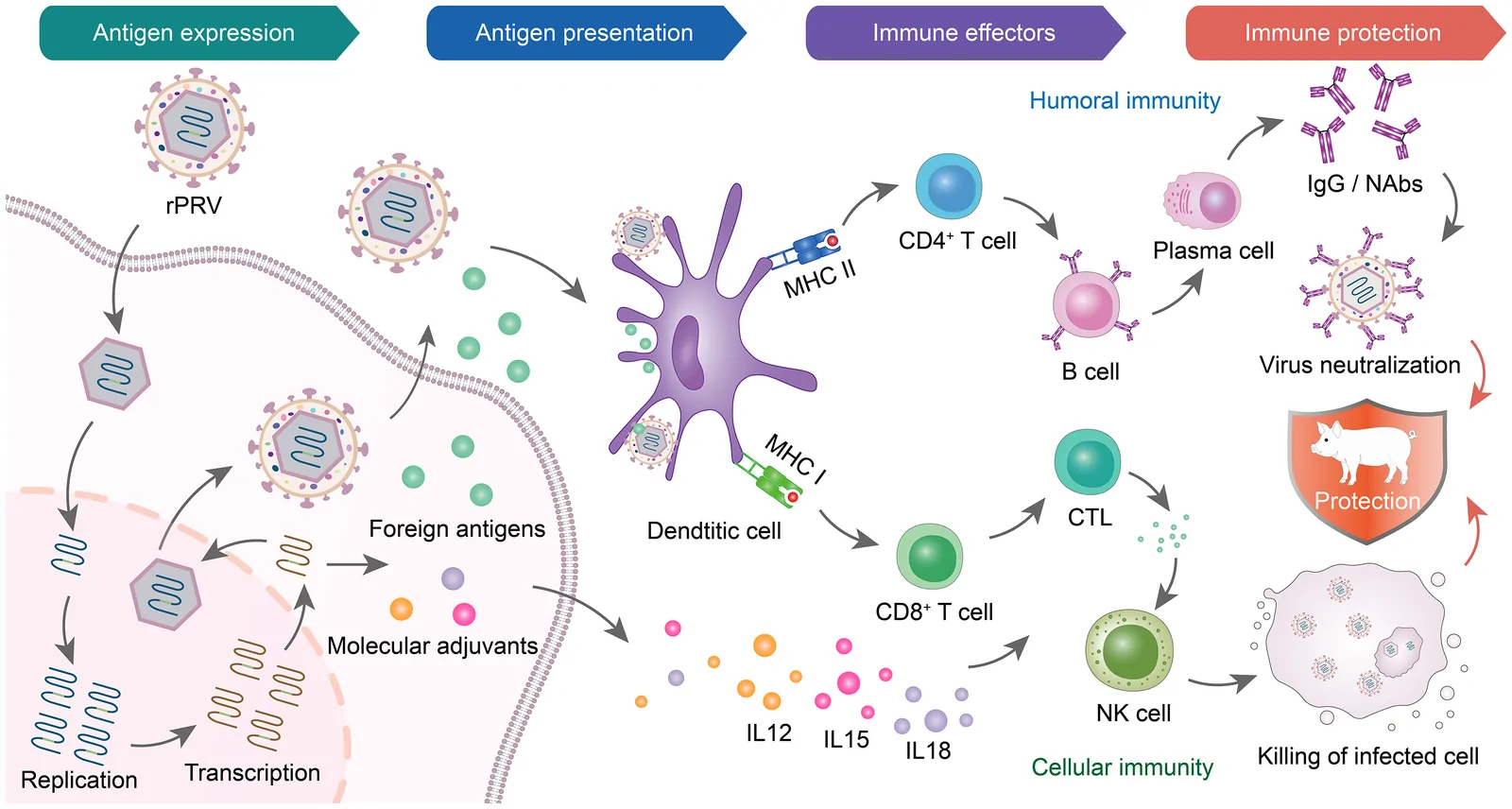
Schematic overview of humoral and cellular immune responses induced by PRV-vectored vaccines. Following immunization, rPRV replicates locally and expresses the inserted foreign antigen. Antigen-presenting cells take up the antigen and present epitopes via MHC class II to CD4^+^ T helper cells and via MHC class I to CD8^+^ CTLs. Activated B cells produce antigen-specific neutralizing antibodies (humoral immunity), while CTLs and Th1-type cytokines (e.g., IFN-γ) mediate the killing of infected cells (cellular immunity). Both arms act in concert to confer protective immunity against the target pathogen.

## 5 Factors limiting the commercialization of PRV vector vaccine

rPRV vaccines have been extensively researched for over three decades. However, a significant number of reported rPRV vaccine candidates have either failed to progress beyond the research stage or remain unavailable on the commercial market. The development of these products has been hindered by several challenges. First, the emergence of PRV variants has rendered vaccine candidates prior to 2011 unsuitable for the current circulating strains, necessitating the development of novel vaccines that target these prevalent strains. Second, although previous studies using animal models have demonstrated immunogenicity and protective effects, it is important to note that the use of mice or guinea pigs may not fully replicate the immune response in pigs. Third, potential interference between different rPRV vaccines within an immunization program should be carefully considered. Simultaneous or overlapping vaccinations with multiple rPRV vaccines can significantly impact the efficacy and dissemination of subsequent vaccinations. Additionally, maternally derived antibodies in piglets may bind to the vaccine antigen to form antigen-antibody complexes, leading to antigen clearance and reduced vaccine efficacy. Therefore, a well-designed immunization schedule, together with the development of markers that differentiate vaccine-induced immunity from natural infection, is essential for the effective monitoring of immune responses. The broad host range of PRV, which includes various domestic and wild animals, requires careful evaluation of the environmental and ecological impacts of releasing rPRV vaccines to ensure safety across a range of species, including goats, dogs, and minks. Finally, maintaining the stability of the immunogenic genes inserted during live vector vaccine development is paramount. The selection of highly immunogenic proteins and highly efficient expression elements, particularly robust promoters, constitutes a crucial foundation for the successful development of recombinant live-vector vaccines.

## 6 Conclusions and future prospects

Over an extended period, traditional vaccines that induce the production of NAbs have been effective in preventing PRV. Nevertheless, the coexistence of PRV with other pathogens, such as PCV, CSFV, PRRSV, and PPV, has become prevalent in contemporary intensive swine breeding systems (20). Consequently, vaccine development strategies have undergone a significant shift. rPRV vaccines have emerged as a promising approach, offering the potential to address the limitations of traditional vaccines, streamline immunization protocols, and improve the efficacy of disease control strategies.

In addition to rPRV vaccines, other viral vectors, such as PRRSV and adenovirus vectors, have also been extensively explored for the expression of foreign proteins (109–113). However, no single vaccine platform is universally superior, and its suitability depends on the target pathogen, host population, and immunization requirements. Compared with PRRSV-based vectors, rPRV offers a large, relatively stable DNA genome with multiple insertion sites (TK, gE/gI, and gG), facilitating multivalent antigen expression. Replication-defective adenoviral vectors also support efficient antigen expression but may be affected by pre-existing anti-vector immunity. Attenuated, replication-competent rPRV vectors can potentially sustain antigen expression and induce robust humoral and cellular immunity with fewer doses; however, maternal antibodies, prior PRV immunity, and biosafety concerns may limit their application. VLP vaccines provide a non-replicating, genome-free alternative, although their immunogenicity and booster requirements depend on formulation. Similarly, mRNA/LNP vaccines enable rapid antigen redesign without viral-vector immunity, but antigen expression is generally transient. DIVA compatibility depends on vaccine design and diagnostic strategies rather than the platform alone. Overall, rPRV vectors may be particularly suitable for multivalent swine vaccination when controlled replication, biosafety, and DIVA requirements can be adequately addressed, whereas non-replicating platforms may be preferable when safety constraints or rapid antigen updating are priorities. These studies effectively summarize the construction and evaluation of rPRV vaccines. In terms of PRV vector vaccine construction, the implementation of the co-transfection assay facilitated the simultaneous introduction of the CRISPR system and the viral genome into identical cells, thereby enhancing the editing capabilities of PRV. The use of two sgRNAs significantly improved the efficiency of non-essential gene knockout, thereby enhancing the recombination efficiency of the PRV genome (31). The substitution of virulence-associated genes with foreign genes has markedly attenuated the pathogenicity of rPRV. However, a comprehensive analysis of the safety of rPRV remains imperative. Notably, DIVA capability is largely preserved in these rPRV constructs: because most are built on gE- and TK-deleted backbones that do not restore gE expression, vaccinated pigs remain anti-gE seronegative and can be distinguished from wild-type-infected animals by commercial gE-ELISA.​ Additionally, the stability of the inserted immunogenic genes is a critical consideration. Strategies, such as enhancing the thermostability of the encoded antigen, as demonstrated by the E2 protein of CSFV, have shown the potential to enhance antibody levels in immunized mice, thereby offering new avenues for immunogen optimization (100). Furthermore, the ability to elicit a robust and long-lasting immune response is another important parameter for vaccine development, which is affected by the vaccine and immunization programs. The immunogenicity and immune response elicited by vaccines are associated with the expression levels of the inserted antigens, which can be enhanced through codon optimization, tandem expression, or utilization of a robust promoter (50, 64). Although a single vaccination with an rPRV-vectored vaccine elicits protective immune responses in animal models (30, 50), it is important to acknowledge that single-dose vaccination may not be efficacious for certain diseases. Consequently, booster vaccinations are often necessary to enhance antibody production and ensure prolonged immunity (61, 68). Furthermore, the selection of immunization routes warrants careful consideration as there is a substantial disparity in the immune responses elicited by different routes. Therefore, future clinical trials should aim to define optimal immunization routes for rPRV-vectored vaccines in target animals.

Although rPRV vaccines offer numerous advantages, there is still room for optimization. With advancements in gene editing technology, modifying the PRV genome has become increasingly feasible. However, certain limitations persist. In clinical applications, attenuated PRV vaccines typically require only a single immunization, whereas most reported rPRV vaccines necessitate two doses for the inserted foreign gene to effectively induce antibody production. Additionally, maternal antibodies in piglets may interfere with the vaccine’s efficacy, highlighting the need for a well-optimized immunization strategy. Addressing these challenges will pave the way for the widespread commercialization and application of rPRV vaccines.
